# Acute Dermal Toxicity and Analgesic Effect of a Capsaicinoid Gel Against Formalin-Induced Pain in Wistar Rats

**DOI:** 10.7759/cureus.101293

**Published:** 2026-01-11

**Authors:** Daniel Chans Mwandah, Kiprotich Joshua, Jimmy Angupale, Hanifah Nantogo, Neeza Timothy, Swase Dominic Terkimbi, Regan Mujinya, Barnabas Atwiine, Tadele Mekuriya Yadesa, Jonans Tusiimire, Patrick Engeu Ogwang

**Affiliations:** 1 Pharmacology and Therapeutics, Mbarara University of Science and Technology, Mbarara, UGA; 2 Pharmacology and Therapeutics, Kampala International University - Western Campus, Ishaka, UGA; 3 Pharmacy, Kampala International University - Western Campus, Ishaka, UGA; 4 Pharmaceutical Sciences, Mbarara University of Science and Technology, Mbarara, UGA; 5 Nursing, Mbarara University of Science and Technology, Mbarara, UGA; 6 Biochemistry, Kampala International University - Western Campus, Ishaka, UGA; 7 Physiology, Kampala International University - Western Campus, Ishaka, UGA; 8 Physiology, Equator University of Science and Technology, Masaka, UGA; 9 Pediatrics and Hematology and Oncology, Mbarara University of Science and Technology, Mbarara, UGA; 10 Pharmacy, Mbarara University of Science and Technology, Mbarara, UGA

**Keywords:** analgesic effect, capsaicinoid gel, capsicum annuum, dermal toxicity, formalin test, wistar rats

## Abstract

Background

Pain is a major public health concern, often inadequately managed due to the limitations and side effects of conventional analgesics. *Capsicum annuum*, widely used in traditional medicine, contains capsaicin, a compound with known analgesic properties. Despite its therapeutic promise, data on the dermal toxicity and efficacy of capsaicin-based topical formulations remain limited.

Objective

The main objective of this study is to evaluate the acute dermal toxicity and analgesic efficacy of a capsaicinoid gel derived from *C. annuum* in Wistar rats.

Methods

An acute dermal toxicity test was conducted in female Wistar rats using OECD guideline 402. Rats received a single dermal application of 5% capsaicinoid gel at 200, 1000, or 2000 mg/kg and were observed for clinical signs over 14 days. Analgesic activity was evaluated using a formalin-induced paw-licking model. Male rats received topical treatments of capsaicinoid gel (100, 200, or 400 mg/kg) or 1% diclofenac gel (positive control), followed by formalin injection. Pain behavior was scored in early (0-10 minutes) and late (15-60 minutes) phases. Hematological and biochemical parameters were assessed after 21 days.

Results

No mortality was observed. Transient dermal and autonomic effects (e.g., erythema, tremors) were noted at higher doses but resolved spontaneously. The gel significantly reduced formalin-induced pain behaviors in both test phases, particularly at higher doses. Hematological and biochemical parameters showed mild, dose-related changes but remained within physiological ranges.

Conclusion

The 5% capsaicinoid gel exhibited promising analgesic effects and an acceptable safety profile in rats. These findings support its potential as a topical analgesic, warranting further formulation optimization and chronic toxicity evaluation.

## Introduction

Pain is defined by the International Association for the Study of Pain (IASP) as an unpleasant sensory and emotional experience associated with actual or potential tissue damage [1]. Although acute pain serves a critical protective function, its persistence often evolves into chronic pain, which can lead to significant physiological, psychological, and socioeconomic consequences [2]. Although acute pain is often transient and responsive to standard interventions, it progresses to a chronic state in approximately 10%-20% of cases, frequently resulting in long-term disability and emotional distress [3]. Chronic pain affects approximately 20% of the global population, with conditions such as low back pain, migraine, and musculoskeletal disorders among the most common causes of long-term disability [3,4]. In African populations, the burden is particularly high, with reported prevalence rates reaching 47% for low back pain and 57% for migraine [5].

Despite its prevalence, pain remains underdiagnosed and poorly managed, especially in low-resource settings. Conventional analgesics, including non-steroidal anti-inflammatory drugs (NSAIDs) and opioids, are limited by adverse effects such as gastrointestinal irritation, renal impairment, tolerance, and risk of dependence [6]. These limitations have prompted a growing interest in alternative therapies, particularly those derived from medicinal plants traditionally used for pain relief.

*Capsicum annuum* L., a member of the Solanaceae family, is widely used in traditional medicine for its analgesic, anti-inflammatory, and antioxidant properties. Its primary bioactive compound, capsaicin, acts by binding to the transient receptor potential vanilloid 1 (TRPV1) receptor, leading to depolarization of sensory neurons and subsequent desensitization with repeated exposure [7,8]. Topical capsaicin formulations have been approved by regulatory agencies such as the U.S. Food and Drug Administration (FDA) for conditions like minor muscle pain, joint pain, and osteoarthritis - typically at concentrations including 0.025%, 0.075%, and 0.1% [9] - and an 8% dermal patch for the treatment of post-herpetic neuralgia.

Despite its therapeutic potential, capsaicin is associated with dose-dependent local adverse effects, including burning sensations, erythema, and neurogenic inflammation [10,11]. Furthermore, while traditional use supports its analgesic claims, standardized data on dermal toxicity, systemic effects, and efficacy - especially at higher concentrations - remain limited.

Given this background, the present study was designed to evaluate both the safety and analgesic efficacy of a 5% capsaicinoid gel formulated from *C. annuum* fruit extract. Using OECD guideline 402, the study assessed acute dermal toxicity in Wistar rats, followed by efficacy testing in a formalin-induced pain model. Both safety and efficacy are important steps in establishing the potential of a given herbal product in preclinical studies. The findings aim to provide foundational data for the development of a safe and effective plant-based topical analgesic. 

## Materials and methods

Ethical approval

All experimental procedures were conducted in accordance with institutional and international guidelines for the care and use of laboratory animals, following the principles of the 3Rs (Replacement, Reduction, and Refinement). Ethical approval was obtained from the Mbarara University of Science and Technology Research Ethics Committee (MUST-REC), approval number MUST-2023-916.

Experimental animals

Adult male and female Wistar rats (150-300 g) were obtained from the animal facility of Kampala International University. Animals were housed under standard laboratory conditions (22 ± 3°C, 30%-70% relative humidity, 12-hour light/dark cycle) with free access to food and water. Female rats were used for the dermal toxicity assessment, and male rats were used for analgesic testing. Animals were acclimatized for at least five days before the experiments. Randomization was done using a random number generator to assign rats to experimental groups.

Plant material and extract preparation

Fresh, mature fruits of *C. annuum* were collected from Kasese District, Uganda, with informed consent from the farm owner. The plant was authenticated by a botanist at Mbarara University of Science and Technology, and a voucher specimen was deposited at the university’s herbarium.

Fruits were shade-dried for 14 days and pulverized into powder. Approximately 200 g of powder was macerated in 1 L of distilled water for 24 hours at room temperature. The mixture was filtered, and the filtrate was concentrated using a rotary evaporator, followed by freeze-drying. The percentage yield was calculated, and the dried extract was stored at 4 ± 2°C until use.

Phytochemical screening

Qualitative phytochemical screening was conducted on the aqueous extract using standard protocols to detect the presence of alkaloids (Mayer’s and Wagner’s tests), flavonoids (Shinoda test), saponins (frothing test), tannins (ferric chloride test), glycosides (Keller-Kiliani test), and reducing compounds.

Preformulation and gel formulation

Preformulation studies were conducted to assess solubility, pH, heat stability, hygroscopicity, and drug-excipient compatibility. Based on these characteristics, a 5% w/w gel formulation of the extract was developed using a Quality by Design (QbD) approach. The gel was prepared using standard pharmaceutical excipients (details withheld for IP (intellectual property) protection) and optimized for viscosity, spreadability, and release properties. The final formulation was packaged in aluminum tubes and stored at room temperature until use.

Acute dermal toxicity study

The study followed OECD guideline 402. Six female Wistar rats were randomly assigned to three dose groups (n = 2/group): 200, 1000, and 2000 mg/kg of the 5% capsaicinoid gel. Fur on the dorsal surface was shaved 24 hours prior. A single dose was applied to approximately 10% of the body surface area and covered with a porous gauze dressing for 24 hours.

Animals were observed for signs of toxicity at 0.5, 1, 2, 4, and 6 hours post-application, then daily for 14 days. Body weight, food/water intake, and clinical signs (respiratory, autonomic, and dermal) were recorded. Control animals received no treatment.

Evaluation of analgesic activity

Analgesic activity was assessed using the formalin-induced paw-licking model as described by Yemitan and Adeyemi [12], with minor modifications. Male rats were randomly assigned to five groups (n = 5/group): Group I, positive control (1% w/w diclofenac gel); Group II, capsaicinoid gel 100 mg/kg; Group III, capsaicinoid gel 200 mg/kg; Group IV, capsaicinoid gel 400 mg/kg; and Group V, negative control (distilled water).

These capsaicinoid gel doses were chosen to include a low dose of 100 mg/kg, a medium dose of 200 mg/kg, and a high dose of 400 mg/kg, as required in toxicity studies based on preliminary dermal toxicity studies, while diclofenac was used as a positive control due to its proven efficacy in inflammatory pain models, including formalin-induced models.

Each topical treatment was applied to the plantar surface 30 minutes before formalin injection. Formalin (0.5%, 0.05 mL) was injected subcutaneously into the right hind paw. Pain behavior was assessed in two phases: Phase I (0-10 minutes), neurogenic pain, and Phase II (15-60 minutes), inflammatory pain.

Pain intensity was scored using a validated behavioral scale [13]: 0 = normal weight bearing, 1 = light paw resting, 2 = paw elevation, and 3 = licking/biting/grooming.

Body weight monitoring and sample collection

Animals were weighed on Days 0, 7, 14, and 21. On Day 21, rats were anesthetized, and blood was collected via cardiac puncture for hematological and biochemical analyses.

Hematological and biochemical analysis

Whole blood was analyzed for red and white blood cell counts, hemoglobin (HGB), hematocrit (HCT), platelet indices, and differential counts, using an automated hematology analyzer. Serum was analyzed for biochemical parameters, including alanine aminotransferase (ALT), aspartate aminotransferase (AST), alkaline phosphatase (ALP), creatinine, urea, glucose, albumin, bilirubin, triglycerides, and amylase, using standard enzymatic colorimetric methods.

Statistical analysis

All data were analyzed using GraphPad Prism version 9.5.1 (GraphPad Software, San Diego, CA, USA). Results are presented as mean ± standard error of the mean (SEM). Group comparisons were made using one-way analysis of variance (ANOVA) followed by Tukey’s post hoc test. A p-value < 0.05 was considered statistically significant.

## Results

Phytochemical composition of capsaicinoid extract

Qualitative phytochemical screening of the aqueous extract of *C. annuum* indicated the presence of alkaloids, glycosides, saponins, flavonoids, and reducing compounds. Tannins, phlobatannins, and anthraquinones were absent (Table 1).

**Table 1:** Results of phytochemical screening of Capsicum annum Keys: '+' denotes present and '-' denotes absent.

Chemical constituents	Aqueous extract
Alkaloids	+
Glycoside	+
Saponins	+
Tannins	-
Flavonoids	+
Reducing compounds	+
Phlobatannins	-
Anthraquinone	-

Acute dermal toxicity

No mortality was observed following a single dermal application of the 5% capsaicinoid gel at doses of 200, 1000, or 2000 mg/kg. However, transient autonomic and dermal reactions were recorded in a dose-dependent manner. These included hyperventilation, lethargy, tremors, and localized erythema and edema, particularly at 1000 and 2000 mg/kg. All observed signs resolved spontaneously within 24-48 hours, and no abnormal behaviors were noted at 7 or 14 days post-application (Table 2).

**Table 2:** Behavioral patterns of rats following capsaicinoid extract treatment

Treatment group	Dose level	Signs of dermal toxicity observed in the treated groups		
		Within 24 hours	7 days	14 days
Capsaicinoid	200 mg/kg	Hyperventilation, mild erythema	All animals are normal	All animals are normal
	1000 mg/kg	Hyperventilation, tremors, lethargy, moderate edema, moderate erythema		
	2000 mg/kg	Dyspnea, hyperventilation, tremors, lethargy, moderate edema, severe erythema		

Analgesic activity in the formalin-induced pain model

The analgesic efficacy study was evaluated in two phases: Phase I (neurogenic pain, 0-10 minutes) and Phase II (inflammatory pain, 15-60 minutes). In both phases, treatment with diclofenac gel (1% w/w) offered the highest protection against pain (50.47 ± 32.23% and 49.85 ± 17.51% for Phase I and II, respectively), followed by treatment with 200 mg/kg capsaicinoid gel (30.86 ± 22.03%) in Phase I and 400 mg/kg capsaicinoid gel (29.71 ± 6.02%) in Phase II. 

The mean pain score in Phase I showed significant group differences (F (4,20) = 5.29, p = 0.005). Animals in the negative control group experienced a significantly higher (p = 0.002, 95% CI: -2.417 to -0.4632) mean pain score compared to those treated with diclofenac gel (1% w/w). Furthermore, in Phase I, there were also significant group differences in the percentage inhibition of pain (F (4,20) = 5.16, p = 0.005). Treatment with diclofenac gel (1% w/w) in Phase I caused a significantly higher (p = 0.003, 95% CI: 0.21-1.55) percentage inhibition of pain compared to no treatment (negative control).

In Phase II, there were significant group differences in both the mean pain score (F (4,20) = 9.99, p = 0.001) and the percentage inhibition of pain (F (4,20) = 7.67, p = 0.006). The study results indicate that there was a significant increase in the mean pain score of animals treated with 200 mg/kg of capsaicinoid gel (p = 0.032, 95% CI: -1.118 to -0.03755) and those in the negative control (p < 0.001, 95% CI: -1.674 to -0.5931) compared to those treated with diclofenac gel (1% w/w). There was also a significant decrease in the mean pain score among animals treated with 100, 200, and 400 mg/kg of capsaicinoid gel (p = 0.019, 95% CI: 0.082-1.162; p = 0.042, 95% CI: 0.0153-1.096; and p = 0.011, 95% CI: 0.1264-1.207, respectively) compared to those in the negative control. Animals in the negative control group had a significantly lower percentage inhibition of pain compared to those treated with diclofenac gel (1% w/w) (p = 0.002, 95% CI: 22.75-76.95) and those treated with 400 mg/kg of capsaicinoid gel (p = 0.027, 95% CI: -56.81 to -2.605) (Table 3). 

**Table 3:** Mean pain scores and percentage inhibition of pain Data are expressed as Mean ± SD, n = 5. * significant p-value when compared to diclofenac group, ^b^ significant p-value when compared with the 100 mg/kg Capsaicin treatment group, ^c^ significant p-value when compared with the 200 mg/kg Capsaicin treatment group, ^d^ significant p-value when compared with the 400 mg/kg Capsaicin treatment group. The control group; w/w represents weight by weight. ANOVA, analysis of variance

Treatment group	Dose (mg/kg)	Phase I		Phase II	
		Mean pain score	Percentage (%) inhibition of pain	Mean pain score	Percentage (%) inhibition of pain
Positive control	Diclofenac gel (1% w/w)	1.4 ± 0.89	50.48 ± 32.23	1.09 ± 0.28	49.85 ± 17.51
Negative control	Not treated	2.84 ± 0.89 *	0.00 ± 0.00 *	2.22 ± 0.19 ^*, b, c, d^	0.00 ± 0.00 ^*, d^
Capsaicinoid extract	100 mg/kg	2.24 ± 0.29	21.24 ± 8.95	1.6 ± 0.32	26.65 ± 22.06
	200 mg/kg	1.96 ± 0.61	30.86 ± 22.03	1.67 ± 0.42 ^*^	25.89 ± 14
	400 mg/kg	2.36 ± 0.26	16.95 ± 8.24	1.56 ± 0.11	29.71 ± 6.02
ANOVA results		F (4, 20) = 5.29; p = 0.005	F (4, 20) = 5.16; p = 0.005	F (4, 20) = 9.99; p = 0.001	F (4, 20) = 7.67; p = 0.006

Body weight changes

Longitudinal body weight monitoring revealed profound sex-dependent responses to topical capsaicinoid gel treatment across the 21-day period. Male rats demonstrated consistent weight gain in all groups without statistically significant differences at any time point (F (3,16) = 0.62, p = 0.615; F (3,16) = 0.81, p = 0.507; F (3,16) = 0.80, p = 0.512; and F (3,16) = 1.18, p = 0.349 for start, Days 7, 14, and 21, respectively). Control males reached 304.20 ± 7.90 g by Day 21, while the 100 mg/kg group showed the lowest final weight (281.68 ± 7.86 g), though this difference remained non-significant (p = 0.349).

Female rats exhibited prominent dose- and time-dependent variations throughout the study period. Significant differences emerged as early as baseline (Day 0) (F (3,16) = 5.08, p = 0.012), with animals in both the 100 mg/kg (246.20 ± 12.11 g) and 400 mg/kg (244.56 ± 12.46 g) capsaicinoid gel treatment groups weighing significantly less than those in the control group (275.78 ± 23.17 g; p = 0.039, 95% CI: 1.341-57.82 and p = 0.028, 95% CI: 2.981-59.46, respectively).

At the end of Day 7, capsaicinoid gel treatment effects became more pronounced (F (3,16) = 7.99, p = 0.002). Animals in the control group (285.44 ± 20.73 g) weighed significantly more than those in the 100 mg/kg (250.28 ± 11.72 g, p = 0.006, 95% CI: 9.437-60.88) and 400 mg/kg (249.18 ± 11.67 g, p = 0.005, 95% CI: 10.54-61.98) capsaicinoid gel treatment groups.

After two weeks (Day 14), treatment with capsaicinoid gel caused significant variations in the body weights of animals (F (3,16) = 17.18, p < 0.001). There was a significant decrease in the body weights of animals treated with 100 mg/kg of capsaicinoid gel (p < 0.001, 95% CI: 29.11-79.93), 200 mg/kg of capsaicinoid gel (p = 0.021, 95% CI: 3.852-54.67), and 400 mg/kg of capsaicinoid gel (p < 0.001, 95% CI: 29.43-80.25) compared to those in the control group. Furthermore, there was also a significant decrease (p = 0.048, 95% CI: 0.1722-50.99) in the body weight of animals treated with 400 mg/kg of capsaicinoid gel compared to those treated with 200 mg/kg of capsaicinoid gel.

At the end of the study (Day 21), animals in the control group reached 326.28 ± 22.06 g, while those in the capsaicinoid gel treatment groups ranged from 258.22 ± 11.57 g (400 mg/kg) to 284.62 ± 11.90 g (200 mg/kg). Animals in the control group had significantly higher body weights compared to those treated with 100, 200, and 400 mg/kg of capsaicinoid gel (p < 0.001, 95% CI: 40.17-95.59; p = 0.003, 95% CI: 13.95-69.37; and p < 0.001, 95% CI: 40.35-95.77, respectively) (Table 4).

**Table 4:** Body weight variations (in grams) of rats throughout 21 day Capsaicin treatment period Data are expressed as Mean ± SD, n = 5. * significant p-value when compared to the control, ^a^ significant p-value when compared with the 100 mg/kg Capsaicin treatment group, ^b^ significant p-value when compared with the 200 mg/kg Capsaicin treatment group (male and females separated). ANOVA, analysis of variance

Treatment duration	Control group	Capsaicin fruit extract dose levels in mg/kg			ANOVA results
		100	200	400	
Males					
Start	272.02 ± 14.34	257.26 ± 7.23	263.86 ± 9.43	269.04 ± 31.82	F (3, 16) = 0.62; p = 0.615
Day 7	283.72 ± 12.35	265.34 ± 6.95	271.9 ± 10.9	276.06 ± 33.86	F (3, 16) = 0.81; p = 0.507
Day 14	292.96 ± 11.05	274.18 ± 7.79	281.92 ± 11.27	282.12 ± 34.33	F (3, 16) = 0.8; p = 0.512
Day 21	304.2 ± 7.9	281.68 ± 7.86	291.72 ± 13.28	287.68 ± 35.2	F (3, 16) = 1.18; p = 0.349
Females					
Start	275.78 ± 23.17	246.2 ± 12.11 ^*^	268.44 ± 11.63	244.56 ± 12.46 ^*^	F (3, 16) = 5.08; p = 0.012
Day 7	285.44 ± 20.73	250.28 ± 11.72 ^*^	274.18 ± 10.24	249.18 ± 11.67 ^*^	F (3, 16) = 7.99; p = 0.002
Day 14	309.98 ± 19.25	255.46 ± 12.95 ^*^	280.72 ± 11.31 ^*^	255.14 ± 11.08 ^*, b^	F (3, 16) = 17.18; p < 0.001
Day 21	326.28 ± 22.06	258.4 ± 13.27 ^*^	284.62 ± 11.9 ^*, a^	258.22 ± 11.57 ^*^	F (3, 16) = 21.96; p < 0.001

Hematological profile

Treatment with capsaicinoid gel induced dose-dependent changes in hematological parameters. In males, significant reductions were noted in red blood cell count (RBC), HGB, and HCT at the highest dose. Similar trends were observed in females at the 200 mg/kg and 400 mg/kg doses. White blood cell (WBC) counts and platelet indices (plateletcrit (PCT) and mean corpuscular volume (MPV)) showed variable modulation across doses, indicating potential systemic effects (Table 5).

**Table 5:** Hematological profile of rats treated with Capsaicin for 21 days Data are expressed as Mean ± SD, n = 5. * significant p-value when compared to the control group, ^a^ significant p-value when compared with the 100 mg/kg Capsaicin treatment group (male and females separated). WBCs, white blood cells; RBCs, red blood cells; HGB, hemoglobin; HCT, hematocrit; MCV, mean corpuscular volume; MCH, mean corpuscular hemoglobin; MCHC, mean corpuscular hemoglobin concentration; PLT, platelets; LYM, lymphocytes; MON, monocytes; GRAN, granulocytes; RDW-CV, red cell distribution width - coefficient of variation; RDW-SD, red cell distribution width - standard deviation; PCT, plateletcrit; MPV, mean platelet volume; PDW, platelet distribution width

Hematological parameters	Control group	Capsaicin fruit extract dose levels in mg/kg			ANOVA results
		100	200	400	
Males					
WBC (10^3^/uL)	8.6 ± 1.92	12.1 ± 1.65	9.38 ± 4.42	8.74 ± 1.63	F (3, 16) = 1.86; p = 0.176
RBC (10^6^/uL)	9.28 ± 2.17	9.36 ± 0.72	9.06 ± 0.69	9.76 ± 0.55	F (3, 16) = 0.29; p = 0.833
HGB (g/dL)	17.42 ± 0.65	17.28 ± 1.11	16.74 ± 0.75	16.82 ± 0.48	F (3, 16) = 0.93; p = 0.451
HCT (%)	51.68 ± 1.88	50.66 ± 3.18	48.42 ± 2.47	49.26 ± 1.77	F (3, 16) = 1.84; p = 0.181
MCV (fL)	50.58 ± 2.62	54.1 ± 1.32	53.62 ± 3.41	50.58 ± 1.15	F (3, 16) = 3.36; p = 0.064
MCH (pg)	17.02 ± 0.72	18.46 ± 0.76	18.58 ± 1.46	17.28 ± 0.58	F (3, 16) = 3.62; p = 0.055
MCHC (g/dL)	33.72 ± 0.36	34.12 ± 1.05	34.56 ± 0.79	34.14 ± 0.49	F (3, 16) = 1.12; p = 0.369
PLT (10^3^/uL)	544.4 ± 24.6	493.8 ± 81.9	464.2 ± 64.1	463.4 ± 75.5	F (3, 16) = 1.69; p = 0.209
LYM (%)	5.08 ± 0.85	8.48 ± 1.43 ^*^	5.88 ± 1.84 ^a^	5.34 ± 1.19 ^a^	F (3, 16) = 6.41; p = 0.005
MON (%)	0.68 ± 0.08	1.12 ± 0.31 ^*^	0.62 ± 0.21 ^a^	0.76 ± 0.19	F (3, 16) = 5.46; p = 0.009
GRAN (%)	2.84 ± 1.02	2.5 ± 0.34	2.88 ± 3.73	2.64 ± 0.68	F (3, 16) = 0.04; p = 0.989
RDW-CV (%)	13.96 ± 1.45	13.3 ± 1.27	12.84 ± 0.66	13.44 ± 0.47	F (3, 16) = 0.97; p = 0.429
RDW-SD (fL)	28.38 ± 4.47	28.8 ± 3.06	27.5 ± 1.53	27.18 ± 1.56	F (3, 16) = 0.33; p = 0.801
PCT (%)	0.386 ± 0.02	0.342 ± 0.05	0.33 ± 0.052	0.32 ± 0.05	F (3, 16) = 1.96; p = 0.161
MPV (fL)	7.04 ± 0.19	6.92 ± 0.16	7.08 ± 0.18	6.92 ± 0.16	F (3, 16) = 1.09; p = 0.379
PDW (%)	15.42 ± 0.89	16.56 ± 0.51	16.4 ± 1.15	16.2 ± 0.68	F (3, 16) = 1.81; p = 0.186
Females					
WBC (10^3^/uL)	8.82 ± 2.16	9.5 ± 3.56	10.58 ± 2.87	11.54 ± 3.82	F (3, 16) = 0.72; p = 0.557
RBC (10^6^/uL)	8.8 ± 0.95	8.29 ± 0.49	8.28 ± 0.83	9.03 ± 0.54	F (3, 16) = 1.31; p = 0.304
HGB (g/dL)	16.08 ± 2.06	15.56 ± 0.45	15.72 ± 1.51	16.38 ± 1.54	F (3, 16) = 0.29; p = 0.827
HCT (%)	45.68 ± 6.55	44.22 ± 1.62	43.94 ± 3.95	46.94 ± 4.52	F (3, 16) = 0.48; p = 0.704
MCV (fL)	51.86 ± 2.15	53.38 ± 1.53	53.12 ± 2.48	51.9 ± 1.95	F (3, 16) = 0.75; p = 0.536
MCH (pg)	18.26 ± 0.60	18.8 ± 1.012	19.02 ± 1.37	18.12 ± 0.74	F (3, 16) = 0.96; p = 0.432
MCHC (g/dL)	35.26 ± 0.52	35.2 ± 0.85	35.78 ± 0.94	34.9 ± 0.47	F (3, 16) = 1.28; p = 0.315
PLT (10^3^/uL)	496.2 ± 89	455.2 ± 53.5	451.6 ± 25.3	571.8 ± 293.7	F (3, 16) = 0.64; p = 0.601
LYM (%)	6.42 ± 2.11	7.2 ± 3.19	6.92 ± 1.85	7.72 ± 2.45	F (3, 16) = 0.25; p = 0.864
MON (%)	1.04 ± 0.27	0.98 ± 0.36	0.96 ± 0.23	1.28 ± 0.46	F (3, 16) = 0.93; p = 0.447
GRAN (%)	1.36 ± 0.42	1.32 ± 0.05	2.7 ± 1.15	2.54 ± 1.14	F (3, 16) = 3.93; p = 0.058
RDW-CV (%)	13.34 ± 0.87	12.46 ± 0.64	12.58 ± 0.94	12.92 ± 1.19	F (3, 16) = 0.9; p = 0.462
RDW-SD (fL)	27.64 ± 1.94	26.62 ± 1.93	26.72 ± 1.63	26.88 ± 3.44	F (3, 16) = 0.19; p = 0.898
PCT (%)	0.362 ± 0.06	0.324 ± 0.04	0.306 ± 0.02	0.416 ± 0.22	F (3, 16) = 0.89; p = 0.464
MPV (fL)	7.34 ± 0.26	7.12 ± 0.21	6.82 ± 0.18	7.48 ± 0.95	F (3, 16) = 1.58; p = 0.234
PDW (%)	16.32 ± 0.81	17 ± 0.48	16.5 ± 0.48	16.08 ± 0.22	F (3, 16) = 2.6; p = 0.088

Biochemical parameters

Biochemical analysis revealed mild alterations in some serum markers after 21 days of dermal exposure. In male rats, serum amylase was significantly elevated at 200 mg/kg and 400 mg/kg (p < 0.05), while glucose levels decreased at the mid-dose. Female rats showed reduced creatinine and elevated glucose levels at higher doses. Liver enzymes (ALT and AST) and renal markers (urea and creatinine) remained within reference limits across all groups (Table 6).

**Table 6:** Biochemical profile of rats treated with Capsaicin for 21 days Data are expressed as Mean ± SD, n = 5. * significant p-value when compared to the control, ^a^ significant p-value when compared with the 100 mg/kg Capsaicin treatment group (male and females separated). AST/GOT, aspartate aminotransferase/glutamate oxaloacetate transaminase; ALP, alkaline phosphatase; ALT/GPT, alanine aminotransferase/glutamate pyruvate transaminase; ANOVA, analysis of variance

Biochemical parameters	Control group	Capsaicin fruit extract dose levels in mg/kg			ANOVA results
		100	200	400	
Males					
Albumin (g/dL)	3.058 ± 0.07	3.028 ± 0.11	2.97 ± 0.24	3.072 ± 0.12	F (3, 16) = 0.48; p = 0.703
ALP (U/L)	107.9 ± 39.85	127.2 ± 48.76	128.2 ± 29.76	142.2 ± 56.2	F (3, 16) = 0.49; p = 0.69
Amylase (U/L)	346.3 ± 67.94	411.8 ± 53.36	506 ± 159.36	499.8 ± 41.39	F (3, 16) = 3.38; p = 0.083
Creatinine (mg/dL)	0.438 ± 0.23	0.564 ± 0.03	0.524 ± 0.07	0.534 ± 0.07	F (3, 16) = 0.95; p = 0.442
Bilirubin (direct) (mg/dL)	0.188 ± 0.23	0.074 ± 0.04	0.068 ± 0.03	0.084 ± 0.04	F (3, 16) = 1.1; p = 0.337
ALT/GPT (U/L)	63.48 ± 19.07	122.92 ± 87.97	105.28 ± 50.86	115.18 ± 34.07	F (3, 16) = 1.19; p = 0.347
Glucose (mg/dL)	98.04 ± 39.09	146.9 ± 69.57	93.58 ± 35.3	136.02 ± 57.72	F (3, 16) = 1.31; p = 0.305
Bilirubin (total) (mg/dL)	0.256 ± 0.09	0.554 ± 0.38	25.288 ± 34.02	0.42 ± 0.14	F (3, 16) = 2.67; p = 0.082
Triglycerides (mg/dL)	57.8 ± 24.23	77.2 ± 28.08	48.36 ± 73.64	100.8 ± 51.69	F (3, 16) = 1.14; p = 0.365
Urea (mg/dL)	35.12 ± 5.46	26.7 ± 4.32	32 ± 3.57	33.6 ± 5.07	F (3, 16) = 3.09; p = 0.057
AST/GOT (U/L)	375.2 ± 224.49	364 ± 169.28	302.4 ± 57.24	399 ± 160.87	F (3, 16) = 0.31; p = 0.815
Females					
Albumin (g/dL)	2.8 ± 0.07	2.984 ± 0.19	3.172 ± 0.29 ^*^	2.898 ± 0.15	F (3, 16) = 3.38; p = 0.044
ALP (U/L)	103 ± 34.05	117.8 ± 40.02	88.2 ± 34.64	124.8 ± 65.23	F (3, 16) = 0.65; p = 0.597
Amylase (U/L)	398.6 ± 58.65	439.6 ± 91.44	430.8 ± 40.96	458.2 ± 51.91	F (3, 16) = 0.77; p = 0.529
Creatinine (mg/dL)	0.608 ± 0.05	0.528 ± 0.06	0.534 ± 0.05	0.542 ± 0.06	F (3, 16) = 2.25; p = 0.122
Bilirubin (direct) (mg/dL)	0.116 ± 0.07	0.07 ± 0.03	0.062 ± 0.03	0.074 ± 0.02	F (3, 16) = 1.52; p = 0.247
ALT/GPT (U/L)	119.4 ± 15.86	101.98 ± 64.79	74.8 ± 17.96	67.2 ± 18.12	F (3, 16) = 2.29; p = 0.117
Glucose (mg/dL)	66.2 ± 36.51	70.36 ± 29.96	171.44 ± 21.96 ^*, a^	162.68 ± 33.24 ^*, a^	F (3, 16) = 17.12; p < 0.001
Bilirubin (total) (mg/dL)	0.456 ± 0.49	0.254 ± 0.05	0.352 ± 0.17	0.266 ± 0.09	F (3, 16) = 0.62; p = 0.615
Triglycerides (mg/dL)	136.8 ± 36.51	87.8 ± 54.18	78.4 ± 24.48	106 ± 49.08	F (3, 16) = 1.82; p = 0.184
Urea (mg/dL)	32.16 ± 3.73	29.58 ± 6.32	29.96 ± 3.94	27.42 ± 7.99	F (3, 16) = 0.57; p = 0.645
AST/GOT (U/L)	366.2 ± 434.63	181.6 ± 23.03	226.2 ± 47.02	201.6 ± 14.22	F (3, 16) = 0.73; p = 0.550

## Discussion

This study evaluated the acute dermal toxicity and analgesic efficacy of a 5% capsaicinoid gel derived from *C. annuum* in Wistar rats. The findings demonstrate that the formulation is non-lethal upon dermal application, produces only transient local and systemic effects, and significantly reduces formalin-induced nociceptive behaviors in a dose-dependent manner.

Phytochemical screening confirmed the presence of bioactive compounds, such as alkaloids, flavonoids, saponins, glycosides, and reducing agents, many of which have been previously associated with anti-inflammatory and analgesic activities [14,15]. The absence of tannins and anthraquinones in the aqueous extract may reduce the potential for dermal irritation and cytotoxicity, which is consistent with the transient and reversible nature of erythema and edema observed in this study.

The acute dermal toxicity test, performed in accordance with OECD guideline 402, showed no mortality even at 2000 mg/kg. Although transient autonomic effects, such as hyperventilation, tremors, and lethargy, were observed at higher doses, these effects resolved spontaneously and did not persist beyond the first 24-48 hours. This suggests that, while the extract may cause temporary stimulation of sensory neurons consistent with capsaicin’s known TRPV1-mediated effects, it does not induce irreversible systemic toxicity [9,10].

The analgesic activity of the capsaicinoid gel was demonstrated in both the neurogenic (Phase I) and inflammatory (Phase II) phases of the formalin test. The strongest inhibition of nociceptive behavior was observed at the 400 mg/kg dose, which significantly reduced paw-licking behavior compared to the untreated control. These results are consistent with the literature, suggesting TRPV1-mediated neuro-inflammatory mechanisms [7,16], though direct demonstration would require specific assays. Previous studies have shown that topical capsaicin leads to functional desensitization of nociceptors, reducing the perception of both thermal and chemical stimuli [17,18].

Interestingly, while body weight increased over time in all animals, those treated with higher doses of capsaicinoid gel exhibited attenuated weight gain. This may reflect mild systemic stress or decreased feeding behavior, secondary to early autonomic effects, though further metabolic and behavioral studies are needed to confirm this.

Hematological and biochemical analyses revealed subtle but significant changes in select parameters. Reductions in RBC, HGB, and HCT values at higher doses could indicate mild suppression of erythropoiesis or increased red cell turnover. However, to clarify potential hematological mechanisms, future studies should include reticulocyte counts (markers of bone marrow response) and iron metabolism. Elevated serum amylase in males, along with altered glucose and creatinine levels in females, suggests a potential sex-specific systemic response, though values remained within physiological limits and did not indicate overt hepatic or renal toxicity. These findings warrant follow-up in sub-chronic or chronic toxicity models to determine long-term systemic effects.

Limitations

Several limitations should be acknowledged. First, histopathological evaluation of major organs was not performed, which would have provided greater insight into potential systemic toxicity. Second, the study only assessed acute exposure; sub-chronic or repeated-dose studies are necessary to evaluate cumulative effects. Third, the mechanism of analgesic action was inferred based on the literature, but not directly assessed through receptor-binding or molecular assays. Finally, the formulation's physicochemical parameters, such as pH, spreadability, and drug release profile, were optimized but not fully reported, which limits reproducibility for future formulation work.

## Conclusions

This study demonstrates that 5% capsaicinoid gel provides significant analgesic effects with an acceptable safety profile in rats, while revealing critical sex-specific responses in metabolic, immune, and growth parameters. The phytochemical analysis revealed a rich profile of bioactive compounds that likely contribute to the observed therapeutic effects, with the absence of certain compounds potentially contributing to the favorable safety profile. The acute toxicity assessment confirmed the formulation’s safety for topical application, with only transient, dose-dependent effects observed at higher concentrations. The formulation shows phase-specific optimal dosing for analgesia, with 200 mg/kg most effective for neurogenic pain and 400 mg/kg for inflammatory pain. The pronounced sexual dimorphism in safety parameters, with females exhibiting significant weight suppression and metabolic alterations, while males showing immune and pancreatic effects, highlights the importance of sex as a biological variable in analgesic development. The absence of significant hepatorenal toxicity supports the formulation’s safety for topical application, while the observed hematological and biochemical changes, though statistically significant, remained within physiological ranges. These findings support the continued development of capsaicinoid-based topical analgesics, particularly for resource-limited settings where conventional analgesics may be inaccessible or contraindicated.

Future research must prioritize sex-specific efficacy evaluation, chronic toxicity assessment, and mechanistic studies to optimize therapeutic applications. The non-linear dose-response observed in females and the phase-specific analgesic patterns suggest that personalized, sex-tailored approaches may maximize therapeutic benefits while minimizing adverse effects. This study contributes to the growing body of evidence supporting sex-specific pharmacology and provides a foundation for developing safer, more effective, plant-based analgesics for diverse patient populations.
